# Crystal structure of [Th_3_(Cp*)_3_(O)(OH)_3_]_2_Cl_2_(N_3_)_6_: a discrete mol­ecular capsule built from multinuclear organothorium cluster cations

**DOI:** 10.1107/S2056989021008914

**Published:** 2021-09-03

**Authors:** Steven P. Kelley, Pokpong Rungthanaphatsophon, Justin R. Walensky

**Affiliations:** a Department of Chemistry, University of Missouri-Columbia, Columbia, MO 65211, USA

**Keywords:** actinide, thorium, cyclo­penta­dien­yl, polynuclear, sandwich complex, crystal structure

## Abstract

An unusually large and structurally complex charge-neutral polynuclear cluster, hexa-μ_2_-azido-di-μ_3_-chlorido-hexa-μ_2_-hydroxido-di-μ_3_-oxido-hexa­kis­(penta­methyl­cyclo­penta­dien­yl)hexa­thorium–diethyl ether–tetra­hydro­furan (1/0.56/1.44), [Th_3_(C_10_H_15_)_6_Cl_3_(N_3_)_6_(OH)_6_O_2_]·0.56C_4_H_10_O·1.44C_4_H_8_O or [Th_3_(Cp*)_3_(O)(OH)_3_]_2_Cl_2_(N_3_)_6_·0.56C_4_H_10_O·1.44C_4_H_8_O (Cp* = [penta­methyl­cyclo­penta­dien­yl])^−^, has been crystallized as a mixed tetra­hydro­furan/diethyl ether solvate and structurally characterized. The mol­ecule contains a number of unusual features, the most notable being a finite yet exceptionally long cyclic metal-azido chain. These rare features are the consequence of both sterically protecting Cp* ligands and highly bridging oxide and hydroxide ligands in the same system and illustrate the inter­esting new possibilities that can arise from combining organometallic and solvothermal *f*-block element chemistry.

## Abstract 2

The reported mol­ecule contains a number of unusual features, the most notable being a finite yet exceptionally long cyclic metal-azido chain. These rare features are the consequence of both sterically protecting Cp* ligands and highly bridging oxide and hydroxide ligands in the same system and illustrate the inter­esting new possibilities that can arise from combining organometallic and solvothermal *f*-block element chemistry.

## Chemical context   

Penta­methyl­cyclo­penta­dienyl (Cp*) ligands have become almost ubiquitous in the organometallic chemistry of the *f*-block elements (Evans & Davis, 2002 ▸). These ligands protect the reactive metal center and allow the solubilization and recrystallization of metal complexes in non-coordinating organic solvents. The actinides in particular have unique chemical properties owing to the participation of *f*-orbital electrons in chemical bonding (Neidig *et al.*, 2013 ▸) and the breakdown of periodic trends in the elements due to relativistic electron motion (Cary *et al.*, 2015 ▸), making organoactinide chemistry an important frontier in fundamental chemistry. However, the general instability of *f*-element organometallic complexes towards air, moisture, and protic solvents has prevented them from being applied in other areas where *f*-elements have been successfully applied, such as the formation of unique extended structures driven by their unusual coordination polyhedra (Burns & Nyman, 2018 ▸, Li *et al.*, 2017 ▸; Rocha *et al.*, 2011 ▸).

Compared to uranium and the lanthanides, the coordination chemistry of thorium has been surprisingly under-investigated. Of the 55,423 entries in the Cambridge Structural Database [Version 2020.3.0 (November 2020); Groom *et al.*, 2016 ▸] containing an *f*-element, only 1,241 contain thorium, and over two-thirds of these have only been reported since 2010. The increased number of Th-containing structures coincides with a renewed inter­est in actinide chemistry in general, and these studies have revealed inter­esting structural features unique to Th-containing compounds such as a strong tendency of Th^4+^ to form high-nuclearity yet discrete mol­ecular complexes and ions (Wilson *et al.*, 2007 ▸; Knope *et al.*, 2011 ▸; Wacker *et al.*, 2019 ▸.)

The title compound of this study was isolated during research using {Th(Cp*)_2_}-based complexes to study novel organic transformations (Tarlton *et al.*, 2020 ▸; Tarlton, Fajen *et al.*, 2021 ▸) and actinide–main-group bonding involving Th^4+^ (Tarlton, Yang *et al.* 2021 ▸; Rungthanaphatsophon *et al.*, 2018 ▸; Vilanova *et al.*, 2017 ▸). It represents an unprecedented case of overlap between organothorium chemistry and the formation of polynuclear oxo-bridged clusters. Spontaneous cluster formation in other, oxygen-free complexes of Th^4+^ with tetrel group elements is explored in a second publication as part of this joint special issue (Kelley *et al.*, 2021 ▸).
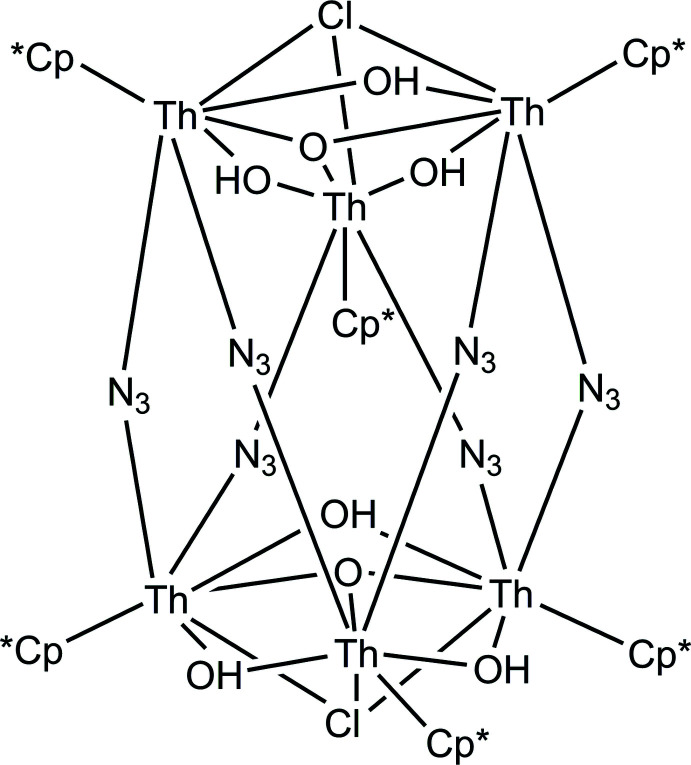


## Structural commentary   

**Figure 1:**
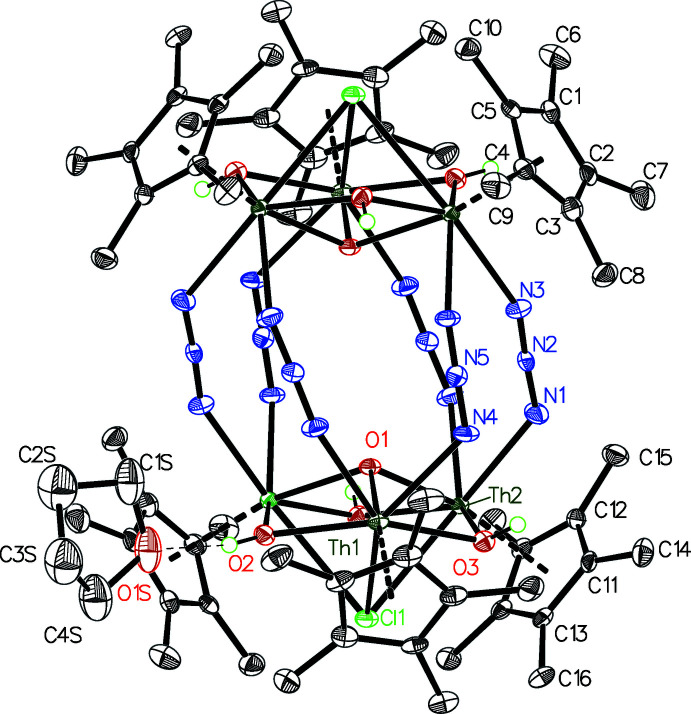
50% probability ellipsoid plot of a single mol­ecule of [Th_3_(Cp*)_3_(O)(OH)_3_]_2_Cl_2_(N_3_)_6._ A tetra­hydro­furan mol­ecule of crystallization is shown to illustrate hydrogen bonding, all other solvents of crystallization are omitted. Hydrogen atoms with the exception of hydroxyl H atoms have been omitted for clarity. Light dashes indicate hydrogen bonding. Unlabeled atoms are symmetry equivalents of labeled atoms.

The mol­ecule is a charge-neutral polynuclear metal complex of unusual size and complexity. It can be conceived as being built from two polyatomic cations with the formula [Th_3_(Cp*)_3_(O)(OH)_3_]^4+^, each of which is sandwiched between two terminal Cl^−^ ions at either end of the mol­ecule and a ring of 6 N_3_^−^ anions in the center (Fig. 1 ▸). The structure crystallizes in the monoclinic space group *C2/m* with *Z* = 2. The mol­ecule resides on a crystallographic mirror plane perpendicular to *b*, a crystallographic twofold proper axis parallel to *b*, and the inversion center where the axis and plane coincide, giving the entire mol­ecule exact *C*_2*h*_ symmetry. All Th^4+^ centers in the structure are chemically equivalent, and the {[Th_3_(Cp*)_3_(O)(OH)_3_]Cl} units have approximate *C*_3*v*_ symmetry. The symmetry of the overall mol­ecule is lowered by the arrangements of the N_3_^−^ ions, which tilt to differing degrees relative to the twofold axis.

There are no published structures containing a moiety exactly analogous to the [Th_3_(Cp*)_3_(O)(OH)_3_]^4+^ cluster, and published Th—O distances vary extremely widely due to the highly variable coordination geometry of Th^4+^. Another neutral hexa­nuclear Th^4+^ complex with the formula Th_6_(O)_4_(OH)_4_(CHO_2_)_12_(OH_2_)_12_ has been reported and shows comparable Th—O^2−^ distances, although the OH^−^ ligands in this structure bridge three metal centers and have significantly longer bond distances (Takao *et al.*, 2009 ▸). A polyatomic anion with the formula [Th_3_Cl_10_(OH)_5_(OH_2_)_2_]^3−^ is reported, which has the same six-membered cycle of Th^4+^ and μ^2^-OH^−^ ligands (but no O^2−^ ligands), and the Th—O distances for these ligands are very similar to those in [Th_3_(Cp*)_3_(O)(OH)_3_]^4+^ (Wacker *et al.*, 2019 ▸).

The six Th^4+^ atoms and six azido ligands are bridged into what is essentially a linear chain that has cyclized to form a 24-membered ring, which is the longest non-polymeric metal–azido chain reported. However, cycles with three or four repeat units are quite common, and one example is known for Th^4+^ and has Th—N and azide N—N distances that overlap with those in this structure (Du *et al.* 2019 ▸). It is clear that while most of the individual building blocks within this structure have been observed previously, the unusual features arise from the termination of growth of a Th^4+^ cluster ion by Cp* ligands, leading to very intricate inter­connectivity between the metal centers.

## Supra­molecular features   

**Figure 2:**
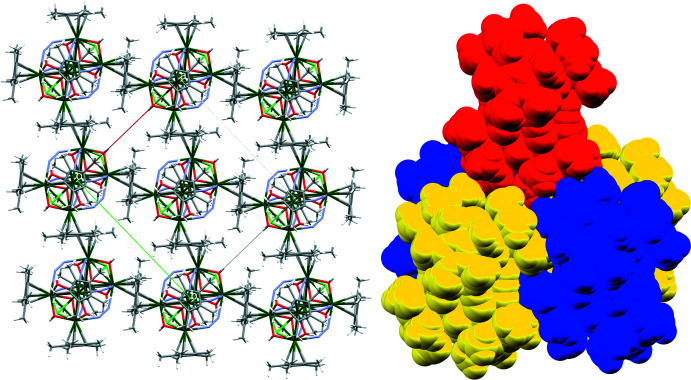
*Left*: Packing diagram showing a single two-dimensional layer of mol­ecules, elements are color coded as in Fig. 1 ▸. *Right:* Packing diagram showing the *CCP*-like arrangement of one mol­ecule (red) over the hole formed by four neighboring mol­ecules in the adjacent layer (yellow/blue, all atoms drawn as spheres of vdW radii).

The mol­ecules pack through a herringbone arrangement of the Cp* ligands so that the methyl groups of one Cp* point towards the aromatic ring plane of the neighboring mol­ecules, leading to infinite two-dimensional layers parallel to the *ab* face (Fig. 2 ▸, *left*). These layers stack along *c* such that the mol­ecules in each layer reside over holes in the neighboring layer, analogous to cubic close packing of spheres (Fig. 2 ▸, *right*); this arrangement most likely reduces repulsion between the like-charged anionic groups at either end of the mol­ecule. For each mol­ecule of the main moiety there are two solvent mol­ecules of crystallization, which are located in the holes in each layer on the crystallographic mirror planes. These solvent mol­ecules were found to be either tetra­hydro­furan (THF) or diethyl ether (Et_2_O) and are substitutionally disordered across the same site; their relative occupancies refined to 72%:28% THF:Et_2_O. Both mol­ecules are positioned such that the ether oxygen atom accepts a hydrogen bond from one of the bridging OH^−^ ions (Table 1 ▸, Fig. 1 ▸).

## Synthesis and crystallization   

The title compound was the byproduct of the reaction of (C_5_Me_5_)_2_Th(CH_3_)[P(Mes)(SiMe_3_)], Mes = 2,4,6-Me_3_C_6_H_2_, (Rungthanaphatsophon *et al.*, 2018 ▸) with two equivalents of Me_3_SiN_3_ in di­meth­oxy­ethane (DME) at room temperature. After stirring overnight, the resulting solution was allowed to crystallize inside an N_2_-filled glove box at ambient temperature (∼3 days). Crystals suitable for SCXRD were obtained by recrystallization of these solids from diethyl ether/tetra­hydro­furan. The chloride is presumably due to the starting material, (C_5_Me_5_)_2_Th(CH_3_)(Cl), which is used to make (C_5_Me_5_)_2_Th(CH_3_)[P(Mes)(SiMe_3_)], while the oxo- and hydroxide ligands are due to an adventitious source of oxygen present in the solvent or glove box.

## Refinement   

Crystal data, data collection and structure refinement details are summarized in Table 2 ▸. The crystal structure was solved by an iterative dual space approach as implemented in *SHELXT* (Sheldrick, 2015*a* ▸). All atoms could be refined anisotropically. The residual difference map contained large, chemically non-reasonable peaks near the Th atoms which could not be modeled but could be reduced by truncating some of the high-angle data during reduction. The disordered THF and Et_2_O mol­ecules were located from the difference map. For the THF mol­ecule, the oxygen atom and carbon atom C1*S* had their *y* coordinates fixed to reside on the crystallographic mirror plane; the other atoms were refined as additionally disordered across both positions related by the mirror plane. All non-hydrogen atoms of the Et_2_O mol­ecule had their *y* coordinates fixed to lie on the mirror plane. The chemical occupancy of the THF mol­ecule was fixed to a free variable, which refined to 72 (1)%, and the chemical occupancies of the THF and Et_2_O mol­ecules were constrained to sum to 100%. The fractional occupancies of all atoms in both solvent mol­ecules were set to 50% of the chemical occupancies due to their residence on or disorder across a crystallographic mirror plane. Both solvent mol­ecules were also refined with C—C distances restrained to 1.54 (2) Å, C—O distances restrained to 1.41 (2) Å, and all anisotropic displacement parameters among bonded atoms restrained to be equal within an e.s.d. of 0.01 Å^2^. A hydrogen atom was located from the difference map for the non-hydrogen bonding –OH group, and its coordinates were refined with the O—H distance restrained to 0.84 (2) Å. For the –OH group engaged in the strong hydrogen bond with THF, a hydrogen atom was placed along the ideal O—H⋯O hydrogen bond vector and restrained to a distance of 0.84 Å from the covalently bonded O atom. The identities of the –OH groups are established on the basis of charge-balance considerations and consistency with Th—O distances in the literature for –OH *vs* O^2−^ ligands, rather than the location of H atoms from the difference map. All other hydrogen atoms were placed in calculated positions, and were constrained to ride on their carrier atoms. Methyl group hydrogen atoms were refined with a riding-rotating model (except for disordered Et_2_O methyl groups which were fixed in idealized staggered geometries). For all H atoms, displacement parameters were constrained to be multiples of *U*_iso_ for the bonded non-hydrogen atom.

## Supplementary Material

Crystal structure: contains datablock(s) I. DOI: 10.1107/S2056989021008914/zl5025sup1.cif

Structure factors: contains datablock(s) I. DOI: 10.1107/S2056989021008914/zl5025Isup2.hkl

CCDC reference: 2105511

Additional supporting information:  crystallographic information; 3D view; checkCIF report

## Figures and Tables

**Table 1:** Hydrogen-bond geometry (Å, °)

*D*—H⋯*A*	*D*—H	H⋯*A*	*D*⋯*A*	*D*—H⋯*A*
O2—H2⋯O1*S*	0.84 (2)	1.93 (3)	2.762 (10)	170 (8)

**Table 2:** Experimental details

Crystal data	
Chemical formula	[Th_6_(C_10_H_15_)_6_Cl_2_(N_3_)_6_(OH)_6_O_2_]·0.56C_4_H_10_O·1.44C_4_H_8_O
*M*_r_	2804.00
Crystal system, space group	Monoclinic, *C*2/*m*
Temperature (K)	100
*a*, *b*, *c* (Å)	17.6783 (14), 17.2647 (14), 16.383 (2)
β (°)	121.867 (3)
*V* (Å^3^)	4246.6 (7)
*Z*	2
Radiation type	Mo *K*α
μ (mm^−1^)	10.59
Crystal size (mm)	0.17 × 0.13 × 0.04

Data collection	
Diffractometer	Bruker VENTURE CMOS area detector
Absorption correction	Multi-scan (*AXScale*; Bruker, 2017 ▸)
*T*_min_, *T*_max_	0.333, 0.431
No. of measured, independent and observed [*I* > 2σ(*I*)] reflections	64622, 5050, 4393
*R*_int_	0.071
(sin θ/λ)_max_ (Å^−1^)	0.650

Refinement	
*R*[*F*^2^ > 2σ(*F* ^2^)], *wR*(*F* ^2^), *S*	0.027, 0.056, 1.03
No. of reflections	5050
No. of parameters	307
No. of restraints	155
H-atom treatment	H atoms treated by a mixture of independent and constrained refinement
	
Δρ_max_, Δρ_min_ (e Å^−3^)	3.14, −0.91

Computer programs: *APEX3* and *SAINT* (Bruker, 2017 ▸), *SHELXT* (Sheldrick, 2015*a* ▸), *SHELXL* (Sheldrick, 2015*b* ▸), and *OLEX2* (Dolomanov *et al.*, 2009 ▸).

## supplementary crystallographic information

### Crystal data

**Table:**

[Th_6_(C_10_H_15_)_6_Cl_2_(N_3_)_6_(OH)_6_O_2_]·0.56C_4_H_10_O·1.44C_4_H_8_O	*F*(000) = 2598
*M**_r_* = 2804.00	*D*_x_ = 2.193 Mg m^−^^3^
Monoclinic, *C*2/*m*	Mo *K*α radiation, λ = 0.71073 Å
*a* = 17.6783 (14) Å	Cell parameters from 9971 reflections
*b* = 17.2647 (14) Å	θ = 2.4–27.5°
*c* = 16.383 (2) Å	µ = 10.59 mm^−^^1^
β = 121.867 (3)°	*T* = 100 K
*V* = 4246.6 (7) Å^3^	Irregular, colorless
*Z* = 2	0.17 × 0.13 × 0.04 mm

### Data collection

**Table:**

Bruker VENTURE CMOS area detector diffractometer	4393 reflections with *I* > 2σ(*I*)
Radiation source: Incoatec IMuS microfocus Mo tube	*R*_int_ = 0.071
shutterless ω and phi scans	θ_max_ = 27.5°, θ_min_ = 2.4°
Absorption correction: multi-scan (*AXScale*; Bruker, 2017)	*h* = −22→22
*T*_min_ = 0.333, *T*_max_ = 0.431	*k* = −22→22
64622 measured reflections	*l* = −21→21
5050 independent reflections	

### Refinement

**Table:**

Refinement on *F*^2^	Primary atom site location: dual
Least-squares matrix: full	Secondary atom site location: difference Fourier map
*R*[*F*^2^ > 2σ(*F*^2^)] = 0.027	Hydrogen site location: mixed
*wR*(*F*^2^) = 0.056	H atoms treated by a mixture of independent and constrained refinement
*S* = 1.03	*w* = 1/[σ^2^(*F*_o_^2^) + (0.0229*P*)^2^ + 34.2882*P*] where *P* = (*F*_o_^2^ + 2*F*_c_^2^)/3
5050 reflections	(Δ/σ)_max_ = 0.003
307 parameters	Δρ_max_ = 3.14 e Å^−^^3^
155 restraints	Δρ_min_ = −0.90 e Å^−^^3^

### Special details

**Table:**

Geometry. All esds (except the esd in the dihedral angle between two l.s. planes) are estimated using the full covariance matrix. The cell esds are taken into account individually in the estimation of esds in distances, angles and torsion angles; correlations between esds in cell parameters are only used when they are defined by crystal symmetry. An approximate (isotropic) treatment of cell esds is used for estimating esds involving l.s. planes.

### Fractional atomic coordinates and isotropic or equivalent isotropic displacement parameters (Å^2^)

**Table:**

	*x*	*y*	*z*	*U*_iso_*/*U*_eq_	Occ. (<1)
Th1	0.68056 (2)	0.60822 (2)	0.69358 (2)	0.01292 (5)	
Th2	0.53556 (2)	0.500000	0.75710 (2)	0.01168 (6)	
Cl1	0.73648 (11)	0.500000	0.86411 (11)	0.0174 (3)	
O1	0.5917 (3)	0.500000	0.6566 (3)	0.0147 (9)	
O2	0.7638 (3)	0.500000	0.6957 (3)	0.0169 (10)	
H2	0.809 (3)	0.500000	0.691 (6)	0.025*	
O3	0.5971 (2)	0.62494 (18)	0.7689 (2)	0.0168 (7)	
H3	0.563 (6)	0.663 (4)	0.748 (7)	0.025*	0.5
N1	0.4076 (3)	0.5858 (3)	0.6357 (3)	0.0233 (9)	
N2	0.3749 (3)	0.5981 (2)	0.5529 (3)	0.0153 (8)	
N3	0.3402 (3)	0.6128 (2)	0.4715 (3)	0.0228 (9)	
N4	0.5440 (3)	0.6925 (2)	0.5842 (3)	0.0198 (9)	
N5	0.500000	0.6919 (3)	0.500000	0.0168 (12)	
C1	0.8493 (3)	0.6738 (3)	0.8077 (4)	0.0204 (10)	
C2	0.8168 (3)	0.7073 (3)	0.7160 (3)	0.0211 (11)	
C3	0.7461 (4)	0.7577 (3)	0.6962 (4)	0.0236 (11)	
C4	0.7349 (3)	0.7564 (3)	0.7756 (3)	0.0208 (10)	
C5	0.7987 (3)	0.7044 (3)	0.8442 (3)	0.0187 (10)	
C6	0.9278 (4)	0.6209 (3)	0.8601 (4)	0.0307 (13)	
H6A	0.918535	0.585471	0.900768	0.046*	
H6B	0.981731	0.651592	0.900265	0.046*	
H6C	0.934610	0.590903	0.813530	0.046*	
C7	0.8561 (4)	0.6980 (4)	0.6544 (4)	0.0332 (14)	
H7A	0.884053	0.646886	0.665711	0.050*	
H7B	0.901031	0.738296	0.670865	0.050*	
H7C	0.808905	0.702819	0.586503	0.050*	
C8	0.6968 (4)	0.8088 (3)	0.6087 (4)	0.0314 (13)	
H8A	0.675395	0.777497	0.550602	0.047*	
H8B	0.736905	0.849200	0.611231	0.047*	
H8C	0.645907	0.832931	0.607281	0.047*	
C9	0.6695 (4)	0.8042 (3)	0.7866 (4)	0.0276 (12)	
H9A	0.611609	0.803876	0.726080	0.041*	
H9B	0.691318	0.857577	0.803166	0.041*	
H9C	0.663160	0.782241	0.837851	0.041*	
C10	0.8162 (4)	0.6901 (3)	0.9437 (4)	0.0260 (12)	
H10A	0.762198	0.701260	0.944310	0.039*	
H10B	0.864643	0.723791	0.989868	0.039*	
H10C	0.833217	0.635783	0.961429	0.039*	
C11	0.4835 (3)	0.4335 (3)	0.8752 (3)	0.0154 (9)	
C12	0.4289 (4)	0.500000	0.8363 (5)	0.0171 (14)	
C13	0.5718 (3)	0.4589 (3)	0.9384 (3)	0.0154 (9)	
C14	0.4549 (4)	0.3503 (3)	0.8574 (4)	0.0260 (12)	
H14A	0.395664	0.346319	0.798828	0.039*	
H14B	0.452683	0.330173	0.912070	0.039*	
H14C	0.497535	0.319910	0.849670	0.039*	
C15	0.3290 (5)	0.500000	0.7689 (5)	0.0279 (17)	
H15A	0.312567	0.464474	0.715426	0.042*	0.5
H15B	0.308772	0.552425	0.743913	0.042*	0.5
H15C	0.300794	0.483101	0.803799	0.042*	0.5
C16	0.6503 (4)	0.4080 (3)	0.9991 (3)	0.0222 (11)	
H16A	0.705180	0.436174	1.017657	0.033*	
H16B	0.646369	0.361634	0.962550	0.033*	
H16C	0.650705	0.392683	1.056974	0.033*	
O2S	0.962 (2)	0.500000	0.674 (2)	0.061 (4)	0.280 (11)
C5S	1.055 (2)	0.500000	0.720 (2)	0.065 (5)	0.280 (11)
H5SA	1.075920	0.546619	0.701666	0.078*	0.140 (5)
H5SB	1.075920	0.453381	0.701666	0.078*	0.140 (5)
C6S	1.091 (3)	0.500000	0.828 (3)	0.064 (6)	0.280 (11)
H6SA	1.156583	0.500000	0.864533	0.097*	0.280 (11)
H6SB	1.070092	0.546347	0.844821	0.097*	0.140 (5)
H6SC	1.070092	0.453653	0.844821	0.097*	0.140 (5)
C7S	0.923 (2)	0.500000	0.573 (2)	0.058 (4)	0.280 (11)
H7SA	0.942803	0.453573	0.554259	0.069*	0.140 (5)
H7SB	0.942803	0.546427	0.554259	0.069*	0.140 (5)
C8S	0.822 (2)	0.500000	0.522 (3)	0.054 (4)	0.280 (11)
H8SA	0.795779	0.500000	0.452448	0.081*	0.280 (11)
H8SB	0.802133	0.453653	0.540462	0.081*	0.140 (5)
H8SC	0.802133	0.546347	0.540462	0.081*	0.140 (5)
O1S	0.8969 (7)	0.500000	0.6553 (7)	0.050 (2)	0.360 (5)
C1S	0.8526 (10)	0.500000	0.5515 (11)	0.055 (3)	0.360 (5)
H1SA	0.805267	0.539887	0.523128	0.066*	0.360 (5)
H1SB	0.825386	0.448814	0.525057	0.066*	0.360 (5)
C2S	0.9243 (12)	0.5179 (12)	0.5292 (15)	0.066 (4)	0.360 (5)
H2SA	0.934339	0.574303	0.528668	0.079*	0.360 (5)
H2SB	0.910796	0.494664	0.467676	0.079*	0.360 (5)
C3S	1.0042 (13)	0.4768 (12)	0.6179 (14)	0.067 (4)	0.360 (5)
H3SA	0.998454	0.419714	0.613336	0.080*	0.360 (5)
H3SB	1.062238	0.492263	0.627273	0.080*	0.360 (5)
C4S	0.9910 (10)	0.510 (2)	0.6976 (15)	0.061 (3)	0.360 (5)
H4SA	1.025462	0.480111	0.758113	0.073*	0.360 (5)
H4SB	1.008272	0.565129	0.710357	0.073*	0.360 (5)

### Atomic displacement parameters (Å^2^)

**Table:**

	*U*^11^	*U*^22^	*U*^33^	*U*^12^	*U*^13^	*U*^23^
Th1	0.01355 (9)	0.01211 (8)	0.01065 (8)	−0.00232 (6)	0.00472 (7)	−0.00054 (6)
Th2	0.01279 (12)	0.01143 (11)	0.01021 (11)	0.000	0.00567 (9)	0.000
Cl1	0.0168 (8)	0.0177 (8)	0.0122 (7)	0.000	0.0039 (6)	0.000
O1	0.015 (2)	0.014 (2)	0.010 (2)	0.000	0.0034 (19)	0.000
O2	0.014 (2)	0.019 (2)	0.017 (2)	0.000	0.008 (2)	0.000
O3	0.0188 (18)	0.0151 (16)	0.0162 (17)	0.0004 (13)	0.0090 (15)	0.0016 (13)
N1	0.021 (2)	0.033 (2)	0.015 (2)	0.0078 (19)	0.0086 (18)	0.0078 (18)
N2	0.014 (2)	0.0136 (18)	0.017 (2)	0.0046 (16)	0.0073 (17)	0.0007 (15)
N3	0.024 (2)	0.029 (2)	0.012 (2)	0.0009 (19)	0.0074 (18)	−0.0004 (17)
N4	0.019 (2)	0.021 (2)	0.011 (2)	0.0002 (17)	0.0022 (18)	0.0013 (16)
N5	0.018 (3)	0.012 (3)	0.019 (3)	0.000	0.008 (3)	0.000
C1	0.019 (3)	0.018 (2)	0.022 (3)	−0.007 (2)	0.009 (2)	−0.0013 (19)
C2	0.024 (3)	0.021 (2)	0.019 (2)	−0.014 (2)	0.011 (2)	−0.007 (2)
C3	0.027 (3)	0.017 (2)	0.022 (3)	−0.009 (2)	0.010 (2)	−0.002 (2)
C4	0.022 (3)	0.017 (2)	0.017 (2)	−0.007 (2)	0.007 (2)	−0.0037 (19)
C5	0.018 (2)	0.019 (2)	0.014 (2)	−0.010 (2)	0.005 (2)	−0.0050 (19)
C6	0.020 (3)	0.037 (3)	0.028 (3)	0.002 (2)	0.008 (2)	−0.005 (2)
C7	0.035 (3)	0.040 (3)	0.029 (3)	−0.017 (3)	0.020 (3)	−0.013 (3)
C8	0.039 (3)	0.025 (3)	0.021 (3)	−0.010 (3)	0.010 (3)	0.003 (2)
C9	0.031 (3)	0.019 (3)	0.027 (3)	−0.003 (2)	0.012 (3)	−0.008 (2)
C10	0.029 (3)	0.026 (3)	0.018 (3)	−0.006 (2)	0.009 (2)	−0.002 (2)
C11	0.018 (2)	0.018 (2)	0.015 (2)	−0.0038 (19)	0.012 (2)	−0.0010 (18)
C12	0.014 (3)	0.027 (4)	0.015 (3)	0.000	0.010 (3)	0.000
C13	0.022 (3)	0.014 (2)	0.012 (2)	0.0000 (19)	0.010 (2)	0.0008 (17)
C14	0.035 (3)	0.022 (3)	0.028 (3)	−0.008 (2)	0.021 (3)	−0.003 (2)
C15	0.017 (4)	0.045 (5)	0.020 (4)	0.000	0.008 (3)	0.000
C16	0.028 (3)	0.021 (2)	0.017 (2)	0.006 (2)	0.011 (2)	0.007 (2)
O2S	0.059 (6)	0.055 (6)	0.085 (6)	0.000	0.050 (6)	0.000
C5S	0.058 (7)	0.057 (7)	0.090 (7)	0.000	0.046 (7)	0.000
C6S	0.055 (9)	0.058 (9)	0.091 (9)	0.000	0.046 (8)	0.000
C7S	0.058 (6)	0.055 (5)	0.084 (6)	0.000	0.053 (6)	0.000
C8S	0.057 (7)	0.051 (6)	0.081 (7)	0.000	0.054 (6)	0.000
O1S	0.052 (5)	0.049 (4)	0.079 (5)	0.001 (5)	0.054 (5)	0.000
C1S	0.058 (6)	0.051 (5)	0.083 (6)	0.003 (5)	0.054 (5)	0.000
C2S	0.063 (6)	0.059 (6)	0.089 (7)	0.012 (5)	0.050 (6)	0.006 (5)
C3S	0.062 (6)	0.062 (6)	0.090 (7)	0.010 (4)	0.050 (6)	0.009 (4)
C4S	0.056 (6)	0.057 (6)	0.088 (6)	0.001 (4)	0.051 (6)	0.002 (4)

### Geometric parameters (Å, º)

**Table:**

Th1—Cl1	3.0589 (12)	C10—H10A	0.9800
Th1—O1	2.307 (3)	C10—H10B	0.9800
Th1—O2	2.367 (3)	C10—H10C	0.9800
Th1—O3	2.388 (3)	C11—C12	1.417 (6)
Th1—N3^i^	2.530 (4)	C11—C13	1.413 (7)
Th1—N4	2.567 (4)	C11—C14	1.500 (7)
Th1—C1	2.791 (5)	C12—C15	1.510 (10)
Th1—C2	2.816 (5)	C13—C13^ii^	1.417 (9)
Th1—C3	2.820 (5)	C13—C16	1.493 (7)
Th1—C4	2.813 (5)	C14—H14A	0.9800
Th1—C5	2.787 (5)	C14—H14B	0.9800
Th2—Cl1	3.0190 (17)	C14—H14C	0.9800
Th2—O1	2.329 (4)	C15—H15A^ii^	0.98 (6)
Th2—O3	2.378 (3)	C15—H15A	0.9800
Th2—O3^ii^	2.378 (3)	C15—H15B	0.9800
Th2—N1	2.553 (4)	C15—H15B^ii^	0.98 (2)
Th2—N1^ii^	2.553 (4)	C15—H15C	0.9800
Th2—C11	2.791 (4)	C15—H15C^ii^	0.98 (4)
Th2—C11^ii^	2.791 (4)	C16—H16A	0.9800
Th2—C12	2.796 (6)	C16—H16B	0.9800
Th2—C13^ii^	2.780 (4)	C16—H16C	0.9800
Th2—C13	2.780 (4)	O2S—C5S	1.415 (19)
O2—H2	0.84 (2)	O2S—C7S	1.411 (19)
O3—H3	0.84 (2)	C5S—H5SA	0.9900
N1—N2	1.180 (5)	C5S—H5SB	0.9900
N2—N3	1.165 (5)	C5S—C6S	1.542 (19)
N4—N5	1.172 (4)	C6S—H6SA	0.9800
C1—C2	1.418 (7)	C6S—H6SB	0.9800
C1—C5	1.415 (7)	C6S—H6SC	0.9800
C1—C6	1.497 (7)	C7S—H7SA	0.9900
C2—C3	1.414 (8)	C7S—H7SB	0.9900
C2—C7	1.505 (7)	C7S—C8S	1.523 (19)
C3—C4	1.415 (7)	C8S—H8SA	0.9800
C3—C8	1.508 (7)	C8S—H8SB	0.9800
C4—C5	1.415 (7)	C8S—H8SC	0.9800
C4—C9	1.507 (7)	O1S—C1S	1.448 (15)
C5—C10	1.511 (7)	O1S—C4S	1.435 (15)
C6—H6A	0.9800	C1S—H1SA	0.9900
C6—H6B	0.9800	C1S—H1SB	0.9900
C6—H6C	0.9800	C1S—C2S	1.525 (16)
C7—H7A	0.9800	C2S—H2SA	0.9900
C7—H7B	0.9800	C2S—H2SB	0.9900
C7—H7C	0.9800	C2S—C3S	1.563 (17)
C8—H8A	0.9800	C3S—H3SA	0.9900
C8—H8B	0.9800	C3S—H3SB	0.9900
C8—H8C	0.9800	C3S—C4S	1.551 (17)
C9—H9A	0.9800	C4S—H4SA	0.9900
C9—H9B	0.9800	C4S—H4SB	0.9900
C9—H9C	0.9800		
			
O1—Th1—Cl1	65.94 (10)	C8—C3—Th1	119.6 (3)
O1—Th1—O2	72.32 (13)	C3—C4—Th1	75.7 (3)
O1—Th1—O3	73.33 (13)	C3—C4—C9	126.1 (5)
O1—Th1—N3^i^	92.85 (14)	C5—C4—Th1	74.3 (3)
O1—Th1—N4	90.95 (13)	C5—C4—C3	107.5 (4)
O1—Th1—C1	147.52 (14)	C5—C4—C9	126.3 (5)
O1—Th1—C2	160.60 (14)	C9—C4—Th1	117.7 (3)
O1—Th1—C3	164.27 (14)	C1—C5—Th1	75.4 (3)
O1—Th1—C4	151.16 (15)	C1—C5—C4	108.5 (4)
O1—Th1—C5	143.48 (14)	C1—C5—C10	125.4 (5)
O2—Th1—Cl1	66.77 (11)	C4—C5—Th1	76.4 (3)
O2—Th1—O3	129.52 (12)	C4—C5—C10	125.8 (5)
O2—Th1—N3^i^	77.23 (15)	C10—C5—Th1	119.2 (3)
O2—Th1—N4	143.70 (14)	C1—C6—H6A	109.5
O2—Th1—C1	82.76 (14)	C1—C6—H6B	109.5
O2—Th1—C2	89.72 (14)	C1—C6—H6C	109.5
O2—Th1—C3	118.37 (14)	H6A—C6—H6B	109.5
O2—Th1—C4	131.14 (14)	H6A—C6—H6C	109.5
O2—Th1—C5	106.72 (15)	H6B—C6—H6C	109.5
O3—Th1—Cl1	65.92 (8)	C2—C7—H7A	109.5
O3—Th1—N3^i^	140.13 (13)	C2—C7—H7B	109.5
O3—Th1—N4	71.49 (12)	C2—C7—H7C	109.5
O3—Th1—C1	109.77 (13)	H7A—C7—H7B	109.5
O3—Th1—C2	125.35 (13)	H7A—C7—H7C	109.5
O3—Th1—C3	104.02 (14)	H7B—C7—H7C	109.5
O3—Th1—C4	77.98 (13)	C3—C8—H8A	109.5
O3—Th1—C5	81.33 (13)	C3—C8—H8B	109.5
N3^i^—Th1—Cl1	142.14 (10)	C3—C8—H8C	109.5
N3^i^—Th1—N4	71.56 (13)	H8A—C8—H8B	109.5
N3^i^—Th1—C1	101.77 (14)	H8A—C8—H8C	109.5
N3^i^—Th1—C2	75.63 (14)	H8B—C8—H8C	109.5
N3^i^—Th1—C3	79.31 (14)	C4—C9—H9A	109.5
N3^i^—Th1—C4	107.60 (14)	C4—C9—H9B	109.5
N3^i^—Th1—C5	123.06 (14)	C4—C9—H9C	109.5
N4—Th1—Cl1	135.67 (10)	H9A—C9—H9B	109.5
N4—Th1—C1	121.10 (14)	H9A—C9—H9C	109.5
N4—Th1—C2	99.86 (15)	H9B—C9—H9C	109.5
N4—Th1—C3	73.69 (14)	C5—C10—H10A	109.5
N4—Th1—C4	77.07 (14)	C5—C10—H10B	109.5
N4—Th1—C5	105.63 (14)	C5—C10—H10C	109.5
C1—Th1—Cl1	85.43 (10)	H10A—C10—H10B	109.5
C1—Th1—C2	29.29 (14)	H10A—C10—H10C	109.5
C1—Th1—C3	48.19 (15)	H10B—C10—H10C	109.5
C1—Th1—C4	48.40 (15)	C12—C11—Th2	75.5 (3)
C2—Th1—Cl1	114.22 (11)	C12—C11—C14	127.5 (5)
C2—Th1—C3	29.07 (15)	C13—C11—Th2	74.9 (2)
C3—Th1—Cl1	127.82 (11)	C13—C11—C12	107.7 (4)
C4—Th1—Cl1	104.78 (10)	C13—C11—C14	124.7 (4)
C4—Th1—C2	48.11 (15)	C14—C11—Th2	117.4 (3)
C4—Th1—C3	29.10 (14)	C11^ii^—C12—Th2	75.1 (3)
C5—Th1—Cl1	79.97 (10)	C11—C12—Th2	75.1 (3)
C5—Th1—C1	29.39 (15)	C11^ii^—C12—C11	108.3 (6)
C5—Th1—C2	48.16 (14)	C11—C12—C15	125.8 (3)
C5—Th1—C3	48.03 (14)	C11^ii^—C12—C15	125.8 (3)
C5—Th1—C4	29.26 (14)	C15—C12—Th2	118.5 (4)
O1—Th2—Cl1	66.47 (11)	C11—C13—Th2	75.7 (3)
O1—Th2—O3	73.12 (9)	C11—C13—C13^ii^	108.1 (3)
O1—Th2—O3^ii^	73.12 (9)	C11—C13—C16	125.7 (4)
O1—Th2—N1	88.99 (13)	C13^ii^—C13—Th2	75.23 (9)
O1—Th2—N1^ii^	88.99 (13)	C13^ii^—C13—C16	126.1 (3)
O1—Th2—C11	155.49 (10)	C16—C13—Th2	118.1 (3)
O1—Th2—C11^ii^	155.49 (10)	C11—C14—H14A	109.5
O1—Th2—C12	166.28 (18)	C11—C14—H14B	109.5
O1—Th2—C13^ii^	144.28 (14)	C11—C14—H14C	109.5
O1—Th2—C13	144.28 (14)	H14A—C14—H14B	109.5
O3—Th2—Cl1	66.73 (8)	H14A—C14—H14C	109.5
O3^ii^—Th2—Cl1	66.73 (8)	H14B—C14—H14C	109.5
O3^ii^—Th2—O3	130.25 (16)	C12—C15—H15A^ii^	109.5 (13)
O3^ii^—Th2—N1^ii^	73.53 (13)	C12—C15—H15A	109.5
O3—Th2—N1^ii^	140.27 (13)	C12—C15—H15B	109.5
O3—Th2—N1	73.53 (13)	C12—C15—H15B^ii^	109.5 (5)
O3^ii^—Th2—N1	140.27 (13)	C12—C15—H15C^ii^	109.5 (9)
O3—Th2—C11	128.42 (12)	C12—C15—H15C	109.5
O3^ii^—Th2—C11	82.80 (12)	H15A—C15—H15A^ii^	77.5
O3—Th2—C11^ii^	82.80 (12)	H15A—C15—H15B	109.5
O3^ii^—Th2—C11^ii^	128.42 (12)	H15A^ii^—C15—H15B^ii^	109.5
O3—Th2—C12	110.75 (9)	H15A—C15—H15B^ii^	34.6
O3^ii^—Th2—C12	110.75 (9)	H15A—C15—H15C	109.5
O3^ii^—Th2—C13^ii^	107.88 (12)	H15A^ii^—C15—H15C^ii^	109.5
O3—Th2—C13^ii^	81.05 (12)	H15A—C15—H15C^ii^	134.9
O3^ii^—Th2—C13	81.05 (12)	H15B—C15—H15A^ii^	34.6
O3—Th2—C13	107.88 (12)	H15B—C15—H15B^ii^	134.9
N1—Th2—Cl1	137.70 (10)	H15B—C15—H15C	109.5
N1^ii^—Th2—Cl1	137.70 (10)	H15B^ii^—C15—H15C^ii^	109.5
N1^ii^—Th2—N1	70.9 (2)	H15B—C15—H15C^ii^	77.5
N1—Th2—C11	107.32 (14)	H15C—C15—H15A^ii^	134.9
N1^ii^—Th2—C11^ii^	107.32 (14)	H15C—C15—H15B^ii^	77.5
N1—Th2—C11^ii^	79.65 (13)	H15C—C15—H15C^ii^	34.6
N1^ii^—Th2—C11	79.65 (13)	C13—C16—H16A	109.5
N1^ii^—Th2—C12	79.86 (15)	C13—C16—H16B	109.5
N1—Th2—C12	79.86 (15)	C13—C16—H16C	109.5
N1^ii^—Th2—C13	107.11 (14)	H16A—C16—H16B	109.5
N1—Th2—C13	126.15 (13)	H16A—C16—H16C	109.5
N1^ii^—Th2—C13^ii^	126.15 (13)	H16B—C16—H16C	109.5
N1—Th2—C13^ii^	107.11 (13)	C7S—O2S—C5S	109 (3)
C11—Th2—Cl1	108.35 (10)	O2S—C5S—H5SA	110.7
C11^ii^—Th2—Cl1	108.35 (10)	O2S—C5S—H5SB	110.7
C11—Th2—C11^ii^	48.60 (19)	O2S—C5S—C6S	105 (3)
C11^ii^—Th2—C12	29.38 (12)	H5SA—C5S—H5SB	108.8
C11—Th2—C12	29.38 (12)	C6S—C5S—H5SA	110.7
C12—Th2—Cl1	127.25 (14)	C6S—C5S—H5SB	110.7
C13^ii^—Th2—Cl1	80.94 (10)	C5S—C6S—H6SA	109.5
C13—Th2—Cl1	80.94 (10)	C5S—C6S—H6SB	109.5
C13—Th2—C11	29.39 (13)	C5S—C6S—H6SC	109.5
C13—Th2—C11^ii^	48.59 (13)	H6SA—C6S—H6SB	109.5
C13^ii^—Th2—C11	48.59 (13)	H6SA—C6S—H6SC	109.5
C13^ii^—Th2—C11^ii^	29.39 (13)	H6SB—C6S—H6SC	109.5
C13^ii^—Th2—C12	48.39 (16)	O2S—C7S—H7SA	109.6
C13—Th2—C12	48.39 (16)	O2S—C7S—H7SB	109.6
C13^ii^—Th2—C13	29.54 (18)	O2S—C7S—C8S	110 (3)
Th1^ii^—Cl1—Th1	75.30 (4)	H7SA—C7S—H7SB	108.1
Th2—Cl1—Th1^ii^	75.86 (3)	C8S—C7S—H7SA	109.6
Th2—Cl1—Th1	75.86 (3)	C8S—C7S—H7SB	109.6
Th1—O1—Th1^ii^	108.19 (18)	C7S—C8S—H8SA	109.5
Th1^ii^—O1—Th2	107.42 (12)	C7S—C8S—H8SB	109.5
Th1—O1—Th2	107.42 (12)	C7S—C8S—H8SC	109.5
Th1^ii^—O2—Th1	104.25 (18)	H8SA—C8S—H8SB	109.5
Th1—O2—H2	127.6 (5)	H8SA—C8S—H8SC	109.5
Th1^ii^—O2—H2	127.6 (5)	H8SB—C8S—H8SC	109.5
Th1—O3—H3	113 (8)	C4S—O1S—C1S	109.7 (12)
Th2—O3—Th1	103.27 (12)	O1S—C1S—H1SA	110.5
Th2—O3—H3	119 (8)	O1S—C1S—H1SB	110.5
N2—N1—Th2	134.0 (3)	O1S—C1S—C2S	106.2 (13)
N3—N2—N1	176.9 (5)	H1SA—C1S—H1SB	108.7
N2—N3—Th1^i^	155.4 (4)	C2S—C1S—H1SA	110.5
N5—N4—Th1	127.7 (3)	C2S—C1S—H1SB	110.5
N4—N5—N4^i^	179.0 (7)	C1S—C2S—H2SA	112.1
C2—C1—Th1	76.3 (3)	C1S—C2S—H2SB	112.1
C2—C1—C6	126.2 (5)	C1S—C2S—C3S	98.2 (15)
C5—C1—Th1	75.2 (3)	H2SA—C2S—H2SB	109.8
C5—C1—C2	107.6 (4)	C3S—C2S—H2SA	112.1
C5—C1—C6	126.0 (5)	C3S—C2S—H2SB	112.1
C6—C1—Th1	118.4 (3)	C2S—C3S—H3SA	112.0
C1—C2—Th1	74.4 (3)	C2S—C3S—H3SB	112.0
C1—C2—C7	126.5 (5)	C4S—C3S—C2S	98.7 (16)
C3—C2—Th1	75.6 (3)	C4S—C3S—H3SA	112.0
C3—C2—C1	107.9 (4)	C4S—C3S—H3SB	112.0
C3—C2—C7	125.3 (5)	O1S—C4S—C3S	101.6 (15)
C7—C2—Th1	120.9 (3)	O1S—C4S—H4SA	111.5
C2—C3—Th1	75.3 (3)	O1S—C4S—H4SB	111.5
C2—C3—C4	108.4 (4)	C3S—C4S—H4SA	111.5
C2—C3—C8	125.1 (5)	C3S—C4S—H4SB	111.5
C4—C3—Th1	75.2 (3)	H4SA—C4S—H4SB	109.3
C4—C3—C8	126.3 (5)		

Symmetry codes: (i) −*x*+1, *y*, −*z*+1; (ii) *x*, −*y*+1, *z*.

### Hydrogen-bond geometry (Å, º)

**Table:**

*D*—H···*A*	*D*—H	H···*A*	*D*···*A*	*D*—H···*A*
O2—H2···O1*S*	0.84 (2)	1.93 (3)	2.762 (10)	170 (8)
